# Dissipation of Triazole Fungicides in Apples

**DOI:** 10.3390/foods14183210

**Published:** 2025-09-15

**Authors:** Tereza Horska, Jitka Stara, Frantisek Kocourek, Leos Uttl, Jingwen Han, Vladimir Kocourek, Jana Hajslova, Zuzana Hanackova, Dana Schusterova

**Affiliations:** 1 Czech Agrifood Research Center, Drnovska 507/73, 161 06 Prague, Czech Republic; tereza.horska@carc.cz (T.H.);; 2 Department of Food Analysis and Nutrition, Faculty of Food and Biochemical Technology, University of Chemistry and Technology, Technicka 1903/3, 166 28 Prague, Czech Republic; 3 Research and Breeding Institute of Pomology Holovousy Ltd., Holovousy 129, 508 01 Holovousy, Czech Republic

**Keywords:** apple, pesticide dissipation, field trials, triazoles, residue, cold storage, dietary risk, LC-MS/MS

## Abstract

Triazole compounds are members of the demethylation inhibitor class of systemic agents used to combat the most widespread apple diseases worldwide, namely apple scab and powdery mildew. The dissipation kinetics of difenoconazole, mefentrifluconazole, myclobutanil, penconazole, tebuconazole and tetraconazole from commercial products in two apple cultivars (Rosana and Selena) were studied over four years. Pesticide residues in the apples were determined using the QuEChERS extraction procedure, followed by liquid chromatography–mass spectrometry analysis. Triazoles applied 84–100 days before harvest dissipated faster than those applied 44–55 days before harvest, with half-lives of 4.0–22.3 days and 7.1–43.9 days, respectively. Except for tebuconazole, all triazoles were found to be well below 30% of the maximum residue levels at the end of the pre-harvest interval, which is mandatory for products in use. The dissipation of five triazoles was evaluated during cold storage over two subsequent years. Residues of difenoconazole, myclobutanil and tebuconazole were detected at levels above 0.01 mg/kg after more than five months. The calculated risk intake values were lower than the established acceptable daily intake and acute reference dose values, indicating that the acute and chronic risks of pesticide exposure from consuming apples are not expected.

## 1. Introduction

The apple (*Malus domestica* Borkh.) is the most widely produced fruit in Czechia. In 2022, apples accounted for 65% of total fruit production [1]. Global apple production increased by 37% between 2010 and 2023, reaching 97 million tonnes in total in 2023. Apple is one of the top three most widely cultivated fruits [2]. However, fungal diseases cause significant economic losses in apple-growing regions worldwide. The most common diseases of apples globally are apple scab (*Venturia inaequalis* (Cooke) G. Winter) and powdery mildew (*Podosphaera leucotricha* (Ellis & Everh.) E. S. Salmon). Apple scab infection typically leads to the development of symptoms such as brown lesions and fruit deformation, as well as the premature dropping of leaves and fruit. It may also lead to considerable storage scab [3,4]. Powdery mildew causes white powdery spots to form on the leaves and stems, reducing photosynthetic efficiency and decreasing apple tree yield [5,6]. In addition to storage scab, the most frequently observed diseases during apple storage are bull’s eye rot (*Neofabraea* spp.), grey mould (*Botrytis cinerea*) and blue mould (*Penicillium* spp.) [7,8,9,10,11].

Effective protection of apples against fungal pathogens still most often depends on the use of synthetic fungicides with different modes of action [12,13]. Fungicide applications are necessary even when growing scab-resistant cultivars due to the risk of resistance breakdown and infection by other fungal diseases [14,15,16]. Azole compounds (triazoles and imidazoles), which are demethylation inhibitors (DMIs), have been among the most commonly used fungicides against apple scab and powdery mildew worldwide for over forty years [5,12,17]. Triazole compounds are frequently used in Czech apple orchards. The Fungicide Resistance Action Committee (FRAC) provides information on the risk of fungicide resistance and how to mitigate it. DMIs belong to the sterol biosynthesis inhibitors (SBIs) class I, FRAC group code 3, with a medium risk of resistance [18]. Azole fungicides interfere with the formation of fungal cell membranes by blocking the synthesis of ergosterol via the inhibition of P450 cytochrome CYP51 [19]. However, other sterol enzymes can also be affected, including aromatase (CYP19), which is responsible for the final stages of the conversion of androgens to oestrogens [20]. Azole fungicides are widely used in agriculture to control crops against fungal pathogens. Frequent use of DMI fungicides has, in many cases, led to the development of resistance [21,22,23]. The widespread use of azole fungicides in agriculture can also increase the risk of resistance developing in *Aspergillus* species, since azole compounds are used to treat human aspergillosis infections [24,25]. Resistance to triazole fungicides in fungal apple pathogens has been documented, with variable impact on fungicide efficacy [26,27,28]. However, the widely used fungicide difenoconazole still exhibits a low resistance frequency [29,30,31]. A low frequency of resistance is also expected for the novel isopropanol–triazole fungicide active ingredient mefentrifluconazole (trade name: Revysol^®^) [19,30,32], which has replaced the recently withdrawn myclobutanil in Europe. Other triazoles approved in the European Union (EU) for crop protection include difenoconazole, penconazole, tebuconazole and tetraconazole [33].

To protect the health of consumers, national pest control programs are usually established to ensure that the level of pesticides in food products remains below the maximum residue levels (MRLs) [34] or the action limits, which are defined as a percentage of the MRL. These limits are also required by EU retailers and supermarket chains. Some require that fruit should contain no more than three to five residues of pesticide active ingredients, each detected at a level higher than 0.01 mg/kg [35]. In Czechia, the levels of pesticide residues in pome fruits grown using the integrated production system must comply with the requirements of Government Regulation No. 80/2023 Coll. [36], which sets the maximum tolerable concentration of residues in/on fruit at 30% of the level set out in Regulation (EC) No. 396/2005 [34].

The main objectives of the study were to evaluate the dissipation kinetics and half-lives of newly registered mefentrifluconazole and five other triazole fungicides (difenoconazole, myclobutanil, penconazole, tebuconazole and tetraconazole) in treated apples, and to evaluate the dissipation of residues of late-applied triazole fungicides during the cold storage of treated apples. The study also aimed to evaluate the previously described relationship between the application dose and the concentration of the active ingredient in apples after application. Based on the results, recommendations for the application of triazole fungicides were provided to meet the requirements for the production of apples with low levels of residue and to reduce dietary risk.

## 2. Materials and Methods

### 2.1. Plant Protection Products for Field Trial

The plant protection products and their application rates are summarised in Table 1. Table S1 in the Supplementary Material provides a list of fungicide products and their use in apple orchards, including pre-harvest intervals and registration against fungal diseases in Czechia. The fungicide preparations were applied using a HARDI lift-mounted sprayer (Exel Industries S.A., Paris, France) at a rate of 500 L/ha. Prior to the initial triazole application, the orchard was treated with copper- (Flowbrix; copper oxychloride 638 g/L) and sulphur- (Sulfurus; sulphur 798.4 g/kg) based products.

### 2.2. Application Rates of Tested Triazoles Active Ingredients

Apple trees of one part of the apple orchard were treated with fungicidal plant protection products according to the program summarised in Table 2. One to three plant protection products were applied at a time. The application doses of the fungicides (Table 1) were used in accordance with the manufacturers’ recommendations for the individual products.

The tested triazole active ingredient rates ranged from 0.030 kg/ha for tetraconazole to a maximum of 0.176 kg/ha for mefentrifluconazole (authorised dose for one application per year) [37] (see Table 1). Difenoconazole was tested during the study in three different products at different application rates per hectare, as shown in Table 1. Registration of the first tested difenoconazole-based product, Embrelia, expired in 2022 as it also contained isopyrazam, which was not authorised in the EU after 8 June 2022 [33]. The second tested pesticide preparation, Score 250 EC, contains only difenoconazole, while the third tested product, Difol, contains folpet as an additional active ingredient. Trials with Talent (myclobutanil) ended in 2021 because myclobutanil was no longer approved in the EU as of 31 May 2021 [33]. However, myclobutanil products are still used in some parts of the world, e.g., the USA [38].

### 2.3. Field Trials and Sampling

The field trials were conducted over a period of four years (2020–2023) at the experimental apple tree plantation (50.086965 N, 14.298240 E), which is located at the Czech Agrifood Research Center (CARC; formerly the Crop Research Institute) in Prague, Czechia. Three northern rows of apple trees (each 147 m long) were used for the experiment. Two Czech apple cultivars resistant to apple scab with similar ripening times and stable regular fruit production (Rosana and Selena) were selected for apple sampling. An insecticide trial was conducted on the same cultivars, but in a different part of the orchard [39]. In 2020 and 2021, the products were applied against apple scab at later dates (at the end of July and on 10 August), except for the tetraconazole-based product in 2020. A combined tebuconazole-based product (Luna Experience 400SC) was used in September to combat storage diseases. In 2022 and 2023, all fungicide products were sprayed in mid- and late June to assess the degradation of triazole residues below 0.01 mg/kg, and to protect against apple scab and powdery mildew. Weather conditions from the initial application of triazoles to the apple harvest were monitored by an agrometeorological station situated near the experimental apple orchard of the Czech Agrifood Research Center (50.085000 N, 14.298333 E). Temperature, precipitation and solar radiation data for particular years are summarised in Figures S1–S4 of the Supplementary Material.

Samples of apple cultivars were collected one or three days after treatment, then at intervals of one to three weeks until harvest. Apple samples were collected from randomly selected apple trees. Apples were picked from different parts of the crown. Three samples of fruit per cultivar were collected each time. Each sample, consisting of fruit weighing approximately 1 kg, was placed in a plastic bag separately and transported in a cooled box to the Metrology and Testing Laboratory at the University of Chemistry and Technology Prague for analysis (see Section 2.6).

### 2.4. Cold Storage and Sampling

During the 2020 and 2021 harvests, the apples were carefully collected in boxes. Approximately 15 kg of apples from each cultivar were transported to the Research and Breeding Institute of Pomology Holovousy for cold storage. The storage chamber temperature was set to +2 °C (±1 °C), and the controlled atmosphere was managed using Storex technology (Storex CA technology, Gravendeel, The Netherlands). Three samples, each weighing approximately 1 kg, were taken from the Rosana and Selena cultivars twice during the storage period. These samples were transported in cooled boxes to the Metrology and Testing Laboratory for further residue analysis (see Section 2.6).

### 2.5. Reagents and Materials for Laboratory Analyses

The list of reagents and certified analytical standards of the six triazoles, together with the internal standard (purity > 95%), used for the analysis of pesticide residues, along with the supplier/manufacturer, is given in Table S2 of the Supplementary Material. Stock solutions of the standards were prepared in acidified (1% formic acid, *v*/*v*) acetonitrile or methanol, depending on solubility, and stored in a freezer at −20 °C, protected from light. Purified water (TOC ≤ 5 μg/L) was prepared using a Millipore Milli-Q system (Millipore, Bedford, MA, USA).

### 2.6. Sample Preparation

The apples were quartered, and the composite laboratory apple samples of each cultivar were cut into small pieces and frozen at −20 °C for 12 h. The prepared samples were homogenised using a laboratory blender and analysed immediately afterwards, or stored frozen and processed within several days. The stability of the selected fungicides in the comminated apples was examined in a stability study conducted on spiked apple homogenates.

Apple extracts were prepared according to the QuEChERS (Quick, Easy, Cheap, Effective, Rugged and Safe) extraction procedure [40]. First, 10 g of the homogenised apple sample was weighed into a 50 mL centrifuge tube, and 10 mL of acetonitrile was added. The tube was then shaken on a vertical laboratory shaker at 1000 strokes per minute for two minutes. Then, 1 g of NaCl and 4 g of MgSO_4_ were added, after which the sample was shaken for one minute. After adding 100 μL of an internal standard solution (5000 ng/mL), the tube was centrifuged for 5 min at 13,000 rcf. An aliquot of the extract was transferred to a vial for liquid chromatography–tandem mass spectrometry (LC–MS/MS) analysis.

### 2.7. LC–MS/MS Analysis of Pesticide Residues

Triazole pesticide analysis in apples was performed using an Agilent 1290 Infinity II liquid chromatograph coupled to a G6495C Triple Quadrupole mass spectrometer (both Agilent Technologies, Santa Clara, CA, USA). Sample separation was performed using a reverse-phase ACQUITY HSS T3 UPLC column (100 mm × 2.1 mm, 1.8 µm; Waters Corporation, Milford, MA, USA). The injection volume was 2 µL. Electrospray ionisation (ESI) was employed in positive ion mode, with the mass analyser operating in dynamic multiple reaction monitoring (MRM) mode. MassHunter Workstation software (version 10.0; Agilent Technologies, Santa Clara, CA, USA) was used for data acquisition and processing. The MRM transitions measured, the retention times and the collision energies for the targeted analytes are given in Table 3. The detailed parameters of the validated LC–MS/MS method (i.e., the LC gradient and ESI source parameters) are described in our previous study [41].

To compensate for matrix effects, a matrix-matched calibration was used for the quantitative analysis of pesticide residues in apples. Matrix calibration standards were prepared by diluting working standard solutions (prepared in acetonitrile containing 1% formic acid, *v*/*v*) with a QuEChERS extract of a blank apple sample from the same cultivar. The blank apple extract was prepared according to the procedure described in Section 2.6.

Information on quality assurance (QA), quality control (QC) and method validation is provided in a previous publication by Schusterova et al. [39]. The performance characteristics of the analytical method for the targeted analytes are summarised in Table 3. Expanded uncertainty was calculated using factor k = 2, which corresponds to a coverage probability of approximately 95%, according to ILAC G17:01/2021.

### 2.8. Data Analysis

#### 2.8.1. Fungicide Residue Dissipation Kinetics and Action Pre-Harvest Intervals

Statistica^®^ software (Language Packs 14.0.0, TIBCO Software Inc., Palo Alto, CA, USA) was used to describe the time trend of fungicide dissipation. The decrease in the residue levels of fungicides was fitted to a first-order kinetic model and calculated using Equation (1):

(1) $$C_{\left(t\right)}=C_{0}\cdot e^{-kt},$$

where *C*_(*t*)_ (mg/kg) is the pesticide residue concentration at time *t* (day), *C*_0_ (mg/kg) is the initial pesticide residue concentration, *k* is the dissipation rate constant (day^−1^).

The half-life Equation (2),

(2) $$t_{1/2}=\frac{\ln2}{k},$$

was used to determine the time (day) required to reduce the concentration by 50%.

The action pre-harvest intervals (APHIs, in day) were calculated using the following equation:

(3) $$APHI=\frac{\left(\ln C_{\left(t\right)}-\ln C_{0}\right)}{k}+\frac{1}{3}t,$$

where *C*_(*t*)_ is the pesticide residue concentration based on the required action threshold, in this case for *APHI*_(30)_ (i.e., 30% of the MRL) and *APHI*_(0.01)_, (i.e., 0.01 mg/kg). To increase the reliability of the APHIs, the calculated time *t* was extended by one-third after taking into account the uncertainty of the evaluation of the kinetic parameters [41]. As far as the calculated *APHI*_(30)_ or *APHI*_(0.01)_ was shorter than the pre-harvest interval (PHI), it was replaced by the established PHI of evaluated pesticide preparations registered for use on apples in Czechia (see Table S1 in Supplementary Material).

The difference in fungicide dissipation between the years 2020 and 2023 was analysed using the non-parametric Kruskal–Wallis test. Fungicide dissipation in the apple cultivars Selena and Rosana was analysed using the Mann–Whitney nonparametric test. The significance level for tests was set at α = 0.05. The differences between cultivars were also evaluated by stating the expanded analytical measurement uncertainty of fungicide residues in apples.

#### 2.8.2. Relationship Between Application Rates of Active Ingredient and the Residue Concentration in Apples

The relationship between application rates of active ingredient (kg/ha) and the residue concentration (mg/kg) in apples after treatment was verified for applied triazoles according to a previously published equation:

(4) $$R_{0}=1.2593\cdot D,$$

where *R*_0_ (mg/kg) is the average residue level in apples collected one day after application and *D* (kg/ha) is a dose of the applied active ingredient [42]. Since the first apple sampling was carried out 1 or 3 days after the pesticide treatment, the residue concentration for each triazole active ingredient, year of trial and apple cultivar was calculated for the day after treatment (*t* = 1 day) using Equation (1). The linear regression model was created using Microsoft Excel (Microsoft Corporation, Redmont, WA, USA).

### 2.9. Calculation of Dietary Exposure to Pesticide Residues

The risk of acute and chronic exposure to pesticide residues from consuming apples was calculated using the Pesticide Residue Intake Model (PRIMo) proposed by the European Food Safety Authority (EFSA) [43]. Residue intake was calculated based on the consumption of fresh, unpeeled apples; therefore, the processing factor was not taken into account.

Acute risk was assessed by calculating the Estimated Short-Term Intake (*ESTI*, in mg/kg bw) parameter. Considering the type of food commodity, this parameter was calculated using the ‘Case 2a’ equation:

(5) $${ESTI}_{case\;2a}=\frac{\;{HR}_{RPC}\cdot \left({(U}_{e}\cdot v\right)+({TDP}_{RPC}-U_{e}))}{BW},$$

where *TDP_RPC_* is the total consumption of apple by the individual subject within a single day (kg), *U_e_* is the edible portion (kg) (*U_e_* = 143 g for apples), *HR_RPC_* is the highest residue estimation in apple samples (mg/kg), *v* is the variability factor (*v* = 7 for apples) and *BW* is the body weight of the individual subject (kg). The short-term dietary exposure of consumers is expressed as a percentage of the Acute Reference Dose (ARfD, in mg/kg bw).

Chronic risk was assessed by calculating the Estimated Daily Intake (*EDI*, in mg/kg bw/day) parameter using Equation (6):

(6) $$EDI=\frac{\;{MC}_{RPC}\cdot {RC}_{RPC}}{BW}$$

where *MC_RPC_* is the mean consumption of apple by the individual subject (kg), *RC_RPC_* is the residue estimation in apple samples (mg/kg) and *BW* is the body weight of the individual subject (kg). The long-term dietary exposure of consumers is expressed as a percentage of the Acceptable Daily Intake (ADI, in mg/kg bw/day).

The consumption data used for the exposure assessment were obtained from the PRIMo, Revision 4 consumption databases reported for the respective primary raw commodity from the SISP04 survey in Czechia for three selected population classes (see Table 4). The 97.5th percentile of consumption distribution was used for the calculations. The health-based guidance values (i.e., ARfD and ADI) are based on the information provided for individual active ingredients [33].

## 3. Results and Discussion

### 3.1. Dissipation of Triazoles from Different Application Dates

**Figure 1:**
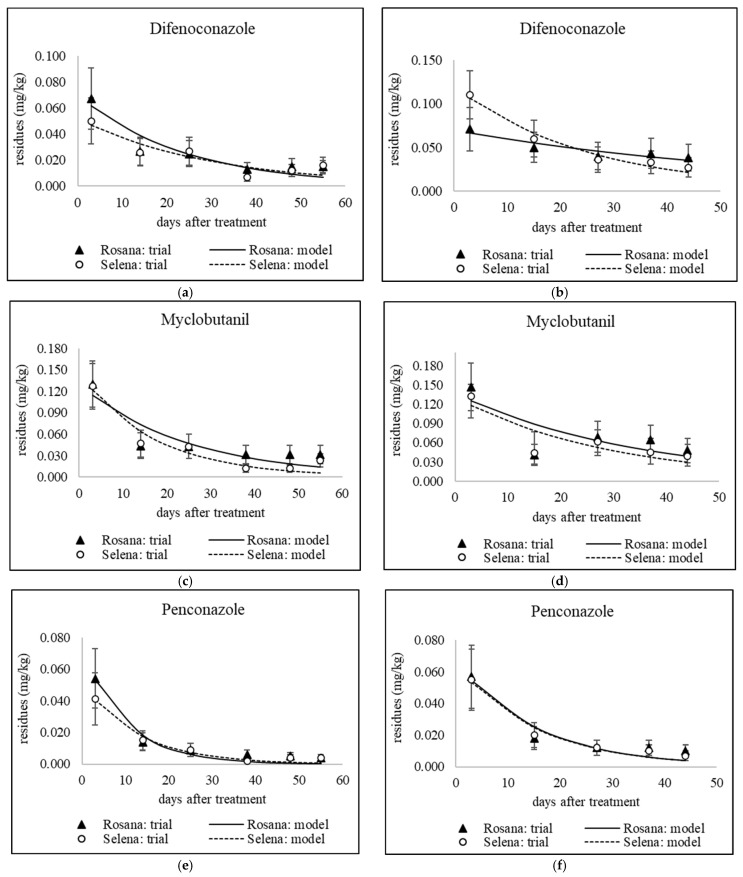
Dissipation of late applied (**a**) difenoconazole (Embrelia); (**c**) myclobutanil; (**e**) penconazoleon on 31 July 2020, and (**b**) difenoconazole (Embrelia); (**d**) myclobutanil; (**f**) penconazole on 10 August 2021. Error bars represent the expanded analytical measurement uncertainty.

**Figure 2:**
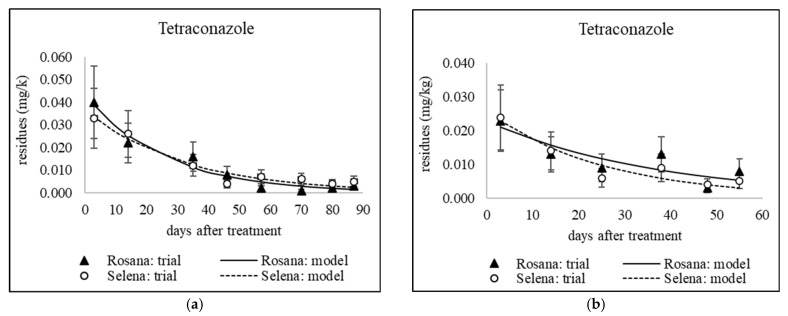
Dissipation of tetraconazole (**a**) early applied on 29 June 2020; (**b**) late applied on 30 July 2021. Error bars represent the expanded analytical measurement uncertainty.

**Figure 3:**
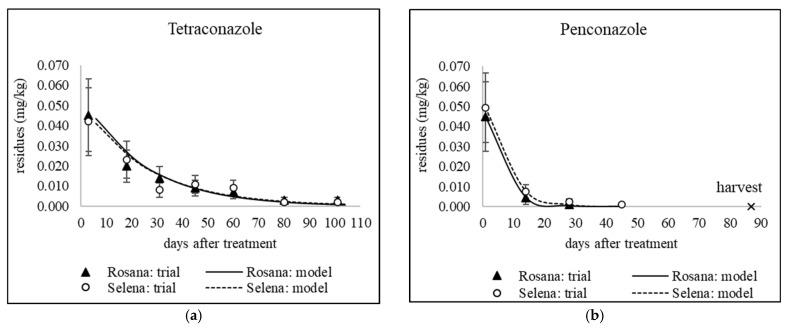
Dissipation of early applied triazoles (**a**) tetraconazole on 13 June 2022; (**b**) penconazole on 26 June 2023. Error bars represent the expanded analytical measurement uncertainty.

**Figure 4:**
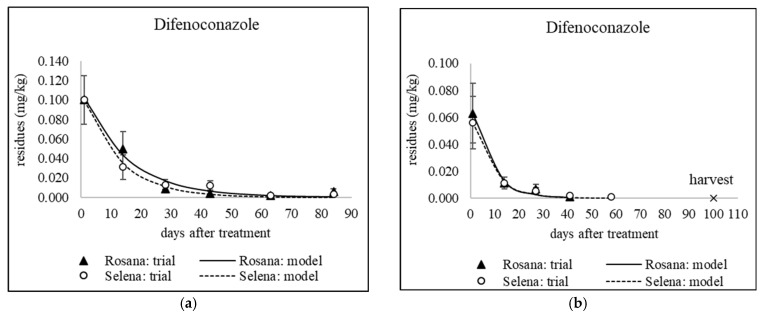

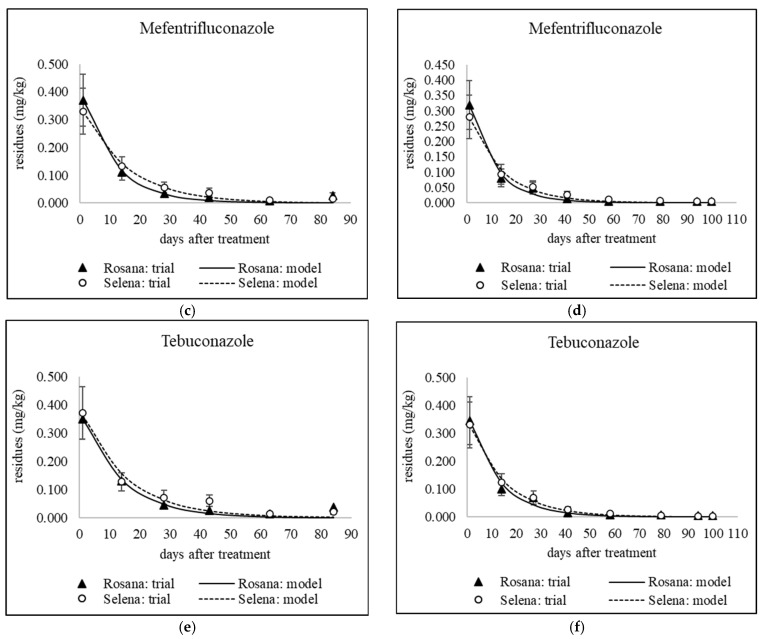
Dissipation of early applied (**a**) difenoconazole (Score 250 EC); (**c**) mefentrifluconazole; (**e**) tebuconazole on 30 June 2022, and (**b**) difenoconazole (Difol); (**d**) mefentrifluconazole; (**f**) tebuconazole on 13 June 2023. Error bars represent the expanded analytical measurement uncertainty.

The decrease in residue levels of the studied fungicides, except tebuconazole applied in September 2020 and 2021 to combat storage diseases, was fitted to a first-order kinetic model (see Table 5). Figure 1, Figure 2, Figure 3 and Figure 4 show sixteen models of triazole dissipation in fruit for each of the cultivars.

Based on the results of the Kruskal–Wallis test, there is a statistically significant difference in fungicide dissipation between years (H = 17.61, *p* < 0.001). Several factors can influence the difference between years, including temperature, photodegradation from sunlight, biodegradation or wash-off [44].

**Figure 5:**
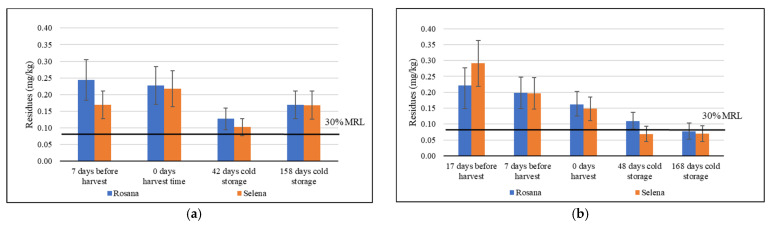
The dynamics of tebuconazole residues in Rosana and Selena apples sampled 3 days after treatment in 2020 and 2021, during ripening in 2021, at harvest time and twice during the cold storage. (**a**) Sampling dates for 2020/21: 17 September, 24 September, 5 November and 1 March; (**b**) sampling dates for 2021/22: 6 September, 13 September, 23 September, 10 November and 10 March. Error bars represent the expanded analytical measurement uncertainty.

**Figure 6:**
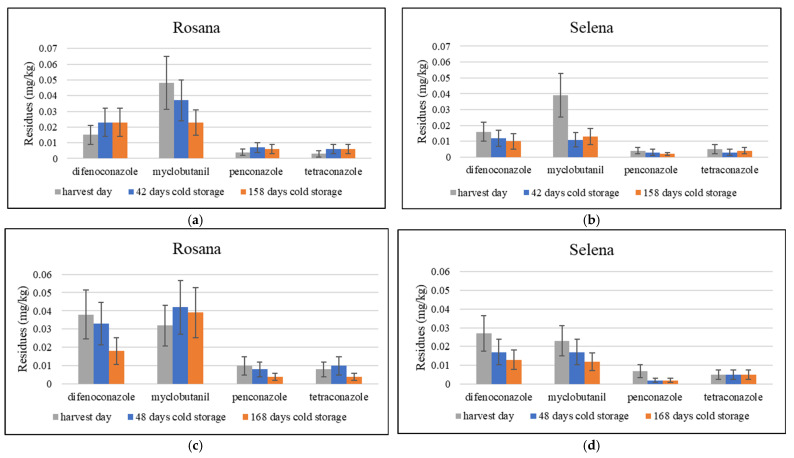
The dynamics of difenoconazole, myclobutanil, penconazole and tetraconazole residues in apple cultivars Rosana and Selena during cold storage. Sampling dates for 2020/21: 24 September, 5 November and 1 March in (**a**) Rosana, (**b**) Selena; sampling dates for 2021/22: 23 September, 10 November and 10 March in (**c**) Rosana, (**d**) Selena. Error bars represent the expanded analytical measurement uncertainty.

A Mann–Whitney U test was conducted to assess the difference in fungicide dissipation half-life between the Rosana and Selena cultivars. The results indicate no statistically significant difference in dissipation rates between the two cultivars (U = 118.5, *p* = 0.726). For individual active ingredients, differences between cultivars were also evaluated for each sampling date using error bars, which represent the expanded analytical measurement uncertainty (see Figure 1, Figure 2, Figure 3 and Figure 4 in Section 3.1 and Figure 5 and Figure 6 in Section 3.2 and Section 3.3). Based on overlapping error bars, no differences in triazole dissipation were found between cultivars. Similar results were obtained for 9 out of 10 insecticides in a previous study by Schusterova et al. [39], where significant differences in dissipation between the Selena and Rosana cultivars were found only for the active ingredient flupyradifurone evaluated in 2021–2023.

#### 3.1.1. Dissipation Dynamics of Triazoles Applied at a Late Stage

Products containing difenoconazole, myclobutanil and penconazole were applied between late July and mid-August, i.e., 44–55 days before harvest. Apple trees were only sprayed with a tetraconazole-based product in late July, 55 days before harvest. The dissipation dynamics of the tested triazoles are shown in Figure 1 and Figure 2b. The dissipation rate constant (*k*) ranged from 0.016 to 0.097 day^−1^ in Rosana and from 0.033 to 0.076 day^−1^ in Selena (see Table 5). Penconazole dissipated fastest in both cultivars, with half-lives in the range of 7.1–10.9 days (see Table 5). However, an experiment in China found that penconazole had a longer half-life in apples of 17.5 days [45]. On the other hand, comparable results were found for other triazoles tested in several published studies. The half-lives of the late-applied myclobutanil were 17.2 and 24.4 days in the Rosana cultivar and 11.8 and 20.6 days in the Selena cultivar, 55 and 44 days before harvest, respectively (see Table 5). The half-lives of myclobutanil applied under temperate conditions in Kashmir, India, at the recommended dosage of 0.125 kg/ha and double the recommended dosage of 0.250 kg/ha were 15.9 and 18.9 days, respectively [46]. The half-lives of tetraconazole applied 55 days before harvest were 17.9 days in the Selena cultivar and 26 days in the Rosana cultivar, as mentioned in Table 5. The half-lives of tetraconazole stereoisomers in apples from three of China’s major apple-producing regions ranged from 11.88 to 39.79 days [47]. The half-lives of difenoconazole applied 55 and 44 days before harvest were 20.8 and 17.5 days, respectively, for the Selena cultivar. For the Rosana cultivar, high variability in difenoconazole half-lives (16.6 and 43.9 days) was observed (see Table 5). A study performed in Poland found that difenoconazole half-lives ranged from 12 to 16 days following a single treatment in early and late June. Two subsequent applications in mid-June and mid-July prolonged the half-life to 21 days [17]. These results suggest that the half-life of 43.9 days observed in Rosana apples, based on a low *k* value of 0.016 day^−1^, is too long and may have been affected by the sampling of apples in 2021. Conversely, no differences were observed between the Selena and Rosana cultivars in terms of the half-lives of penconazole and myclobutanil, which were applied on the same date as difenoconazole in 2021 (see Table 2).

#### 3.1.2. Dissipation Dynamics of Triazoles Applied Early

Difenoconazole, mefentrifluconazole, tebuconazole and tetraconazole were applied in mid- and late June, corresponding to approximately 85 and 100 days before harvest (see Figure 2a, Figure 3a, Figure 4 and Figure 5). Apple trees were treated with penconazole only in late June (87 days before harvest) due to the expected rapid dissipation (see Figure 3b). The dissipation rate constant (*k*) varied from 0.039 to 0.175 day^−1^ in the Rosana cultivar and from 0.031 to 0.141 day^−1^ in the Selena cultivar. Penconazole dissipated the fastest, with half-lives of 4.0 and 4.9 days for the Rosana and Selena cultivars, respectively, following treatment at the end of June. The half-lives after late application were almost twice as long (see Section 3.1.1 and Table 5). Half-lives of less than 12 days were also observed for early-applied difenoconazole (5.7–10.4 days), mefentrifluconazole (7.5–10.8 days) and tebuconazole (8.7–10.7 days) in both cultivars. Tetraconazole showed slower dissipation, with half-lives ranging from 17.2 to 22.3 days in both cultivars, similar to that observed after late application at the end of July (see Section 3.1.1 and Table 5).

The half-life of difenoconazole in apples, as determined in a study conducted across three locations in China, ranged from 6.3 to 10.2 days [48] and are in agreement with our results (Table 5). For pears, the half-life of difenoconazole applied in June and July of 2020–2022 varied from 8.6 to 16.3 days [41].

No studies on the dissipation of mefentrifluconazole in apples have been published so far, and only a few recent studies have focused on its dissipation in field crops [49,50,51]. One field trial was conducted to investigate the efficacy and dissipation of mefentrifluconazole and pyraclostrobin in mangoes in China. The half-life of mefentrifluconazole in mangoes ranged from 5.6 to 10.8 days [50]. Data on the dissipation of mefentrifluconazole from crops such as watermelon and rice are not comparable with the results for apples, and our results provide the first dissipation models for this substance in apples in a temperate climate zone.

In contrast, the dissipation dynamics of tebuconazole were studied in apples [52,53,54,55,56], but the results varied and may have been influenced by weather conditions during the experiment, application rates and timing of application before harvest. The residue dissipation of tebuconazole could be strongly influenced by precipitation, as described in a numerical dynamic model for pesticide residues [44]. Studies focusing on tebuconazole applied late, 15–40 days before harvest, showed widely varying half-lives ranging from 7.7 to 28.86 days [52,53,54]. No half-lives were published for tebuconazole applied early on, but residues below 0.01 mg/kg were found more than 100 days after treatment when it was applied in late June or early July [55,56]. The dissipation of tetraconazole from a study conducted in China [47] is discussed in Section 3.1.1.

#### 3.1.3. Low-Residue Production

The residues of triazoles from all trials were compared with their respective MRLs (Regulation (EC) No. 396/2005) and expressed as a percentage of the MRL (see Tables S3–S6 in the Supplementary Material). The residue concentration of tebuconazole exceeded 30% of the MRL (i.e., 0.090 mg/kg) 14 days after treatment in 2021, 2022 and 2023 (see Tables S4–S6 in the Supplementary Material). The tebuconazole residue concentration calculated for a 14-day PHI in 2022 and 2023 ranged from 0.121 to 0.155 mg/kg (40.3–51.7% of the MRL), meaning that the APHI_(30)_ had to be prolonged to approximately one month (see Table 5).

Residue levels of other triazoles were below the MRL from the first sampling (see Tables S3–S6 in the Supplementary Material). Except for tebuconazole, which was applied to combat post-harvest diseases (see Section 3.3), the residue concentrations of all triazoles were found to be well below 30% of the MRL at harvest time and are therefore suitable for low-residue production (see Tables S3–S6 in the Supplementary Material).

Similarly, a study on insecticide dissipation, carried out on the same apple cultivars, showed that the concentrations of eight of the ten insecticides were below 30% of the MRL (i.e., 2–26% of the MRL) at the end of their pre-harvest interval. Only flonicamid (with metabolites according to the residue definition) and pirimicarb exceeded the limits for low-residue production [39], with concentrations calculated at 28–39% and up to 60% of the MRL, respectively.

#### 3.1.4. Non-Residue Production

Residue levels of 0.01 mg/kg or below at harvest were achievable for penconazole and tetraconazole when applied at the recommended dates. Similar observations were found in a study focused on the production of safe baby food, where trace levels of penconazole (0.005 mg/kg) were detected 33 days after pesticide treatment when it was applied 96 days before harvest, and tetraconazole was found at concentrations below 0.01 mg/kg from 56 to 105 days after application [56]. When applied 44 days before harvest, the residue level for the Rosana cultivar was 0.01 mg/kg. For baby food production, an interval of 50 days before harvest would be safer than the 39.5 days predicted by the model (Table 5). In the case of early difenoconazole application in late June 2022 (84 days before harvest), the 0.01 mg/kg limit was maintained (see column C_(harvest)_ in Table 5 for more details). Our results are consistent with the recommendation of Szpyrka et al. [17] for the application of difenoconazole, as a period of three months is required before harvest to achieve a concentration of 0.01 mg/kg, which is the mandatory limit for the production of baby food.

By contrast, mefentrifluconazole and tebuconazole, which were applied at the end of June 2022, were found to exceed the limit of 0.01 mg/kg in harvested apples. Higher residues of mefentrifluconazole and tebuconazole at harvest were related to a higher applied dose compared to difenoconazole. The residues of difenoconazole, mefentrifluconazole and tebuconazole applied in mid-June 2023 were below 0.01 mg/kg at harvest time (see column C_(harvest)_ in Table 5 for more details). In mid-June, apples are less than 40 mm in diameter (73–74 BBCH), and the dilution of pesticides due to fruit growth is high [44].

Except for tetraconazole, the calculated APHI_(0.01)_ values from the model varied according to the date of application. APHI_(0.01)_ values were proposed for a longer period after application of the later-applied triazoles than the earlier-applied ones. For early-applied difenoconazole-based products, such as Score 250 EC and Difol, the calculated APHI_(0.01)_ values were shorter than the PHI values (see the numbers in brackets in Table 5). However, the PHI is mandatory. In all other triazole experiments, the PHIs were prolonged. The calculated APHI_(0.01)_ values for mefentrifluconazole and tebuconazole, applied in late June 2022, were shorter than the 88-day trial PHI values for both cultivars. However, the residues observed in the harvested apples exceeded 0.01 mg/kg (see Table 5). Paušič et al. [35] similarly detected tebuconazole residues exceeding 0.01 mg/kg in harvested apples 85 and 89 days after the last application of a tebuconazole-based product, and Ticha et al. [56] detected tebuconazole residues above 0.01 mg/kg 83 days after treatment. However, no tebuconazole residues were found in harvested apples 119 days after treatment.

### 3.2. The Dynamics of Tebuconazole Residues from Pre-Harvest Application to Cold Storage

Tebuconazole is the only triazole registered in the two-compound pesticide product Luna Experience 400 SC for use against a complex of storage diseases in Czechia [37]. The fungicide preparation, which has a PHI of 14 days, was applied 10 and 20 days before harvest in 2020 and 2021, respectively (Figure 5). Residues of tebuconazole were detected three days after application at concentrations below the MRL (<0.3 mg/kg). At the time of harvest, the residue concentration in the two cultivars was found to correspond to 72–76% and 49–54% of the MRL in 2020 and 2021, respectively. This indicates that an interval of four days, shorter than the mandatory 14-day PHI, as well as extending the PHI to 20 days, was insufficient to reduce tebuconazole residues to below 30% of the MRL (see Figure 5 and Tables S3 and S4 in the Supplementary Material). The slow dissipation may have been caused by dry weather in September, combined with apple ripening.

Two trials on the dissipation of fluopyram and tebuconazole from apples were conducted in Poland, 15 and 21 days after application [53,54]. The same fungicide product (Luna Experience 400 SC; 0.75 l/ha) was used, as was the MRL of 0.3 mg/kg. In the 2016 trial, tebuconazole residues in three apple cultivars were below 30% of the MRL after 14 days (11.7–27.4% of the MRL) [53], whereas in the 2017 and 2018 experiments, residues exceeded 30% of the MRL even 21 days after application (35–53% of the MRL) [54]. The faster dissipation of tebuconazole during the 2016 field trial could be explained by the higher temperatures during the experiment compared to those in our experiment (see Figures S1 and S2 in the Supplementary Material) and in the trials conducted by Podbielska et al. [54] in 2017 and 2018, which were about 5 °C lower.

Residues of tebuconazole above 30% of the MRL were also detected in the Selena and Rosana cultivars 14 days after the early application of tebuconazole (see Section 3.1.3). However, the use of tebuconazole against apple scab and powdery mildew is not problematic due to the long pre-harvest interval. Conversely, post-harvest disease control products should be applied no later than one month before harvest, meaning that the use of Luna Experience 400 SC for low-residue apple production cannot be guaranteed.

A slight decrease in tebuconazole residues was observed during the cold storage of apples. During the 2020/21 trials, the requirements of Government Regulation No. 80/2023 Coll. on maximum residue levels were not met, even after 158 days (Figure 5a). Conversely, in the 2021/22 trials, the 30% MRL for tebuconazole was reached for the Selena apple cultivar after 48 days of cold storage, and for both tested cultivars after 168 days (Figure 5b). Only a few studies have focused on the fate of pesticides in pome fruit during long-term storage [55,57,58,59]. The effect of low-temperature storage on fungicides was evaluated in pome fruit treated with fungicides several days before harvest for apples and several hours before harvest for pears. It was confirmed that low temperatures significantly slow down the dissipation of pesticide residues [57,58]. The average half-lives of captan, boscalid, pyraclostrobin and trifloxystrobin in apples stored in a controlled atmosphere for 147 days were approximately 170 days [57]. Similarly, the half-lives of tebuconazole at different dilutions were 14.1–15.4 days and 106.6–128.3 days in pears stored at 25 °C and 4 °C, respectively [58].

### 3.3. The Fate of Residues of Difenoconazole, Myclobutanil, Penconazole and Tetraconazole During Cold Storage

Changes in the residue levels of the fungicides difenoconazole, myclobutanil, penconazole and tetraconazole during cold storage were evaluated (Figure 6). Penconazole and tetraconazole residues were found to be below or equal to 0.01 mg/kg at harvest and during cold storage. In contrast, late-applied difenoconazole and myclobutanil residues were variable, often exceeding 0.01 mg/kg even after more than five months in cold storage (Figure 6).

The effect of storage temperature on difenoconazole was evaluated in pears treated several hours before harvest. The half-lives of difenoconazole at different dilutions were found to be 7.5–21.1 days and 44.0–135.1 days in pears stored at 25 °C and 4 °C, respectively [58]. The dissipation of myclobutanil was studied in apples that were treated five times from the beginning of fruit development (20–25 mm) until the ripening stage. Myclobutanil residues were then evaluated at harvest and after two months of storage at 4 °C. Myclobutanil dissipation in the fruit slowed down after harvest during storage [59].

### 3.4. Relationship Between Triazole Application Rates and Residue Concentrations in Apples

**Figure 7:**
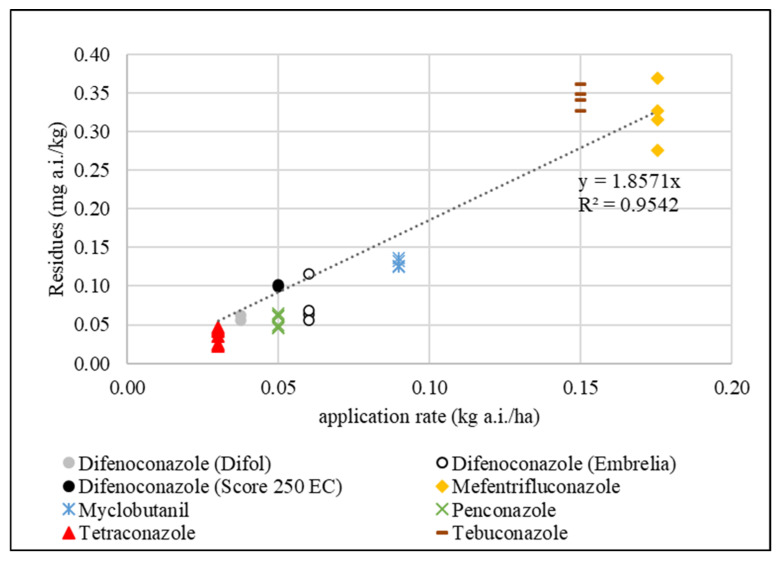
Residue level in apples (C_1_), day after application (y-axis) and the application rate of active ingredients (x-axis) of the tested triazole fungicides.

Due to the wide range of application rates of the tested triazoles, the correlation between application rates (kg/ha) and residue concentrations (mg/kg) in apples after application was verified for six triazoles in eight different product formulations (0.037–0.176 kg/ha), as shown in Table 1. The calculated triazole residue concentrations (C_1_) in apples one day after application (t = 1) correlate well with the application rate via the linear function y = 1.8571x; R^2^ = 0.9542 (see Figure 7). The numeric value of 1.8571 for the triazoles was higher than the value of 1.2593 computed after the application of six different fungicides (captan, pyraclostrobin, trifloxystrobin, boscalid, cyprodinil and fludioxonil) belonging to four different chemical groups, one day after treatment in an apple orchard in Poland [42]. It should be noted that none of the fungicides used to develop the model equation belonged to the triazole group. These differences could be due to the use of different sprayers and/or the application of fungicides to apples at different stages of development. In our experiments, the highest concentrations in apples were found one day after tebuconazole application (0.330–0.370 mg/kg). Two studies from Poland evaluated the dissipation of tebuconazole in apples [53,54]. The same plant protection product and application rate were used, and the average tebuconazole residue the day after treatment ranged from 0.295 to 0.397 mg/kg. These findings confirm that the average tebuconazole concentration after application may be higher than that predicted by the general formula [42].

### 3.5. Dietary Risk Assessment

The risk of exposure to pesticide residues from consuming apples was assessed for Czech consumers based on consumption data for the respective unprocessed commodity, broken down by three population classes. Significant variations were observed in the contributions of different triazole pesticides to both the ARfD and the ADI. Taking all exposure results into account, the highest acute and chronic dietary exposure to pesticide residues was estimated for the ‘other children’ population group, while the lowest dietary exposure was estimated for the ‘adults’ group (Table 6 and Table S7 in the Supplementary Material).

The highest acute exposure estimation (9.033 g/kg bw, corresponding to 31.1% of the ARfD) was observed for tebuconazole in the ‘other children’ population class, which was applied in 2021 against post-harvest diseases (see Table 6A and Table S7A in the Supplementary Material). Conversely, the toxicological contribution of tebuconazole to the adult population was calculated to be in the range of 0.2–11.1% of the ARfD. Although residue concentrations in apples at harvest in 2020 were higher than in 2021, apples were harvested four days before the end of the PHI. In contrast, exposure estimation to other triazole fungicides did not exceed 1.5% of the ARfD in any of the monitored population classes (see Table 6A). A recently published study evaluated the dietary risks of 57 active ingredients found in Polish apples over 17 years. The highest acute risk observed for tebuconazole, at a maximum concentration of 0.5 mg/kg, was 140.1% of the ARfD for toddlers and 36.5% of the ARfD for adults [60]. Conversely, the highest observed residue concentration of difenoconazole (0.027 mg/kg) did not exceed 27% of the ARfD for toddlers and 6.8% of the ARfD for adults [60].

As with the acute risk assessment, the highest estimated long-term intake of residues was found for tebuconazole. Chronic exposure to this fungicide was calculated to be 0.2–12.9% of the Acceptable Daily Intake (ADI) for the ‘other children’ population class, 0.1–5.8% for the ‘adolescent’ population class and 0.05–3.0% for adults. According to the obtained data, the toxicological contribution of the other triazole residues did not exceed 9.1% of the ADI (see Table 6B and Table S7B in the Supplementary Material). The average residue concentrations of 0.09 mg/kg (difenoconazole) and 0.041 mg/kg (tebuconazole) determined over the 17-year study were used for chronic exposure of children and adults to triazoles [60]. According to the presented data, long-term exposure to residues of difenoconazole and tebuconazole in apples was low for 14 children and 16 adult European subpopulations. The highest exposure was found in German children (2.25% ADI for difenoconazole and 0.83% ADI for tebuconazole) and German women (0.46% ADI for difenoconazole and 0.17% ADI for tebuconazole) [60].

### 3.6. Risks Associated with Overuse of Azoles

The azole family of fungicides/antifungals, i.e., triazoles and imidazoles, are used in many areas. They are used in agriculture, for the conservation of wood as biocides, as industrial colorants and in cosmetics [24]. Azoles are also used in human medicine to control fungal pathogens such as *Aspergillus* spp. and *Candida auris* [24,25]. The risks associated with the use of azoles arise from agriculture, the environment, the quality of water sources and the health of people, in terms of dietary risks and the risk of fungal pathogens developing resistance. The utilisation and overuse of azoles should be regulated through technological innovation and legislation. However, the benefits of using azoles, mainly in agriculture and human medicine, far outweigh their potential or real risks.

Azoles are used in agriculture to protect crops against fungal pathogens. Their use reduces yield losses, improves product quality and reduces the incidence of contaminants such as mycotoxins in products. Azole fungicides are frequently used against fungal pathogens affecting wheat, potatoes, sugar beet and oilseed rape, among others. Overuse of azole fungicides leads to the development of resistance in fungal pathogens. In the control of apple, the incidence of resistant *Venturia inaequalis* was observed [29,31,61]. The FRAC has formulated three recommendations for the use of DMIs against apple scab:

- DMI fungicides should not be used for the entire season; a maximum of four DMI sprays, either alone or in a mixture, are recommended.
- DMIs at the recommended label rates should be used in mixtures or block alternations with a non-cross-resistant fungicide.
- Preventative applications should always be the first choice with DMIs. Curative applications are only recommended when accurate disease warning systems are in place [62].

Decreased sensitivity to triazoles and strong cross-resistance between mefentrifluconazole, difenoconazole and tebuconazole were observed in European *Zimoseptoria tritici* populations [63]. Azoles can negatively impact natural enemies such as bees, particularly when used in a tank mix with insecticides [64,65]. Fungicides belonging to the triazole chemical group are usually metabolised via similar pathways through metabolic reactions such as hydroxylation or hydrolysis. These metabolic pathways commonly result in the formation of triazole derivative metabolites (TDMs), including triazole lactic acid, triazole alanine, triazole acetic acid and 1,2,4-triazole [66,67]. An important environmental risk associated with azoles is the presence of 1,2,4-triazole in water, primarily in groundwater and drinking water [68,69,70]. The health risks associated with azoles are low with regard to dietary risks from consuming fruit with azole fungicide residues from integrated apple production (i.e., with residual levels below 30% of the MRL). The highest risk of triazole residues in apples was found for the ‘other children’ category when tebuconazole was applied before harvest to combat storage diseases in apples (see Section 3.5). This finding is consistent with the highest levels of tebuconazole residues in apples reported in a Polish study, where these values exceeded 100% of the ARfD for toddlers [60]. The incidence of triazole residues in food products is regulated by legislation and trade practices. Maintaining MRLs [34] and pre-harvest intervals for pesticides reduces the risk of triazole residues in products. Recently, the incidence of residues in fruit has decreased. During the period 2010–2021, residues of triazoles in apples were below the MRL in EU/EEA countries. Exceeding the ARfD was recorded for tebuconazole in peaches and pears [43]. The levels of pesticide residues in pome fruits grown using the integrated production system in Czechia must comply with the requirements of Government Regulation No. 80/2023 Coll., which sets the maximum tolerable concentration of residues in/on fruit at 30% of the MRL set out in Regulation (EC) No. 396/2005. Similar limits are required by retail chains. To reduce the risks associated with the overuse of azoles, EFSA published information on the impact of azoles on the development of *Aspergillus* spp. resistance and provided recommendations to mitigate the risks to human health [24].

## 4. Conclusions

The dissipation of six triazole fungicides—difenoconazole, mefentrifluconazole, myclobutanil, penconazole, tebuconazole and tetraconazole—after treatment of two apple cultivars was monitored. No significant differences in triazole dissipation were found between Selena and Rosana cultivars. Those applied early (i.e., 84–100 days before harvest) dissipated faster, with half-lives ranging from 4.0 days (penconazole) to 22.3 days (tetraconazole). In contrast, those applied at later stages (44–55 days before harvest) showed longer half-lives, ranging from 7.1 days (penconazole) to 43.9 days (difenoconazole). Among the triazoles evaluated, penconazole dissipated most rapidly regardless of the date of application (half-life ranging from 4.0 to 10.9 days), while tetraconazole dissipated more slowly after both early and late application (half-life from 17.2 to 26.0 days). Tebuconazole was the only triazole found to exceed 30% of the maximum residue level at the end of the pre-harvest interval, meaning its registered use against post-harvest disease cannot be recommended for low-residue apple production. When tebuconazole was applied in September, its residues exceeded this threshold in apples at harvest and even during long-term storage. No significant changes in the content of other tested triazoles were observed during storage.

In addition, residues at or below 0.01 mg/kg were reliably achieved at harvest for penconazole and tetraconazole in all trials, regardless of the chosen application date, and for difenoconazole at early application dates.

The calculated risk intake values were significantly lower than the established ADI and ARfD values, indicating that acute and chronic risks from pesticide exposure through apple consumption are not expected when triazole fungicides are applied according to Good Agricultural Practice. Conversely, the potential adverse effects of increased triazole fungicide use in agriculture have recently been discussed. These include the emergence of resistance in target organisms, as well as cross-resistance to medical azoles in human pathogenic fungi, such as *Aspergillus* spp.

Another issue that has arisen in recent years is the presence of the degradation product 1,2,4-triazole in not only groundwater, but also surface water and drinking water. These issues require further study, and the recommendations issued by FRAC [62] and EFSA [24] must be followed to ensure the continued safe use of triazole products in crop protection.

## Figures and Tables

**Table 1:** Application rate of plant protection products.

Trade Name of PPP ^a^	Active Ingredient (a.i.)	Dose of PPP (kg, L/ha)	Content of Triazole a.i. in PPP (g/kg, g/L)	Dose of Triazole a.i. (kg/ha)
Embrelia ^b^	difenoconazole + isopyrazam	1.5	40.0	0.060
Score 250 EC	difenoconazole	0.2	250	0.050
Difol	difenoconazole + folpet	3.5	10.7	0.037
Belanty	mefentrifluconazole	2.34 ^d^	75.0	0.176
Talent ^c^	myclobutanil	0.45	200	0.090
Topas 100 EC	penconazole	0.5	100	0.050
Domark 10 EC	tetraconazole	0.3	100	0.030
Luna Experience	tebuconazole + fluopyram	0.75	200	0.150

^a^ PPP—plant protection products; product expiry date in Czechia ^b^ 8 September 2022; ^c^ 10 August 2021 [37]; ^d^ authorised dose for one application per year.

**Table 2:** Application dates for triazole-based products and harvest dates in 2020–2023.

Year of Trial		2020	2021	2022	2023
PPP	Active Ingredients	Application Date			
Embrelia	difenoconazole	31 July	10 August	×	×
Score 250 EC		×	×	30 June	×
Difol		×	×	×	13 June
Belanty	mefentrifluconazole	×	×	30 June	13 June
Talent	myclobutanil	31 July	10 August	×	×
Topas 100 EC	penconazole	31 July	10 August	×	26 June
Domark 10 EC	tetraconazole	29 June	30 July	13 June	×
Luna Experience	tebuconazole	14 September	3 September	30 June	13 June
Harvest date		24 September	23 September	22 September	21 September

× Not applied.

**Table 3:** LC–MS/MS method parameters and validation parameters for the targeted analytes.

Analyte	ESI Mode	RT ^a^ (min)	MS/MS Transitions (Collision Energy (V))	LOQ ^b^ (mg/kg)	REC ^c^ (%)	RSD ^d^ (%)	ME ^e^ (%)	Linearity (mg/kg)
difenoconazole	ESI+	9.7 + 10.2	406.0 > 251.0 (24) 408.0 > 253.0 (24)	0.001	81–87	2–9	5	0.001–0.1
mefentrifluconazole	ESI+	9.7	398.1 > 182.1 (36) 398.1 > 70.1 (24)	0.002	95–97	4–5	5	0.002–0.1
myclobutanil	ESI+	8.1	289.1 > 125.0 (40) 289.1 > 70.1 (20)	0.001	92–98	2–8	3	0.001–0.05
penconazole	ESI+	9.4	284.0 > 159.0 (36) 284.0 > 70.1 (16)	0.001	85–86	5–8	−8	0.001–0.05
tebuconazole	ESI+	9.5	308.1 > 125.0 (44) 308.1 > 70.1 (26)	0.002	86–89	1–6	−9	0.002–0.1
tetraconazole	ESI+	8.6	372.0 > 159.0 (44) 372.0 > 70.1 (20)	0.002	82–87	2–4	3	0.002–0.05

^a^ Retention time; ^b^ limit of quantification; ^c^ recovery (mean values of two spiking levels (0.002 and 0.02 mg/kg)); ^d^ relative standard deviation for recovery; ^e^ matrix effect ((A_MSTD_/A_STD_ − 1) × 100%, where A_MSTD_/A_STD_ is the ratio of the peak area of the analyte in the matrix/solvent standard).

**Table 4:** Consumption data of apples in Czechia [43].

Risk Assessment	Population Class ^a^	Mean BW ^b^ (kg)	Unit Weight Consumed (g)
ACUTE	other children	26.3	272.50
	adolescents	45.5	450.00
	adults	74.7	409.50
CHRONIC	other children	26.0	535.00
	adolescents	45.6	319.75
	adults	75.2	385.55

^a^ Other children: 3–10 years old, adolescents: 10–18 years old, adults: 18–65 years old, ^b^ BW: body weight of the individual subject.

**Table 5:** Kinetic model parameters (C_0_, k), dissipation half-lives (t_1/2_), calculated residue concentrations at the end of the pre-harvest interval (C_(model PHI)_) and determined residue concentrations at harvest (C_(harvest)_).

					Kinetic Model Parameters								
Year (Trial PHI)	Cultivar	Active Ingredient	PHI	^a^ MRL	C_0_	k	t_1/2_	R^2^	C_(model PHI)_	30% MRL	C_(harvest)_	APHI_(30%)_	APHI_(0.01)_
(Day)			(Day)	(mg/kg)	(mg/kg)	(Day^−1^)	(Day)		(mg/kg)			(Day)	(Day)
2020 (55)	Rosana	Difenoconazole (Embrelia)	21	0.8	0.070	0.042	16.6	0.8718	0.029	0.240	0.015	21	61.9
	Selena				0.052	0.033	20.8	0.8376	0.026		0.016		65.9
2021 (44)	Rosana				0.070	0.016	43.9	0.8571	0.050		0.038		164.3
	Selena				0.120	0.040	17.5	0.9696	0.052		0.027		83.6
2022 (84)	Rosana	Difenoconazole (Score 250 EC)	49		0.109	0.066	10.4	0.9803	0.004		0.006	49	(47.9) 49
	Selena				0.107	0.080	8.7	0.9853	0.002		0.003		(39.5) 49
2023 (100)	Rosana	Difenoconazole (Difol)	110		0.071	0.122	5.7	0.9907	<0.001		<0.001	110	(21.4) 110
	Selena				0.063	0.115	6.0	0.9942	<0.001		<0.001		(21.2) 110
2022 (84)	Rosana	Mefentrifluconazole	28	0.4	0.404	0.090	7.7	0.9905	0.033	0.120	0.027	28	54.9
	Selena				0.349	0.064	10.8	0.9927	0.058		0.015		73.6
2023 (100)	Rosana				0.346	0.092	7.5	0.9913	0.026		0.006		51.1
	Selena				0.297	0.072	9.6	0.9903	0.040		0.006		62.8
2020 (55)	Rosana	Myclobutanil	14	0.6	0.129	0.040	17.2	0.7788	0.074	0.180	0.032	14 ^b^	84.6
	Selena				0.145	0.059	11.8	0.9230	0.064		0.023		60.6
2021 (44)	Rosana				0.136	0.028	24.4	0.5562	0.092		0.048		122.5
	Selena				0.130	0.034	20.6	0.7279	0.081		0.039		101.8
2020 (55)	Rosana	Penconazole	14	0.15	0.071	0.097	7.1	0.9590	0.018	0.045	0.004	14	26.8
	Selena				0.051	0.076	9.1	0.9752	0.017		0.004		28.3
2021 (44)	Rosana				0.066	0.064	10.9	0.9181	0.027		0.010		39.5
	Selena				0.065	0.064	10.8	0.9673	0.026		0.007		38.9
2023 (87)	Rosana				0.054	0.175	4.0	0.9997	0.005		<0.001		14.0
	Selena				0.057	0.141	4.9	0.9982	0.008		<0.001		16.5
2020 (87)	Rosana	Tetraconazole	14	0.3	0.044	0.039	18.0	0.9625	0.025	0.090	0.003	14	50.9
	Selena				0.037	0.031	22.3	0.9549	0.024		0.005		56.1
2021 (55)	Rosana				0.023	0.027	26.0	0.7390	0.016		0.008		41.2
	Selena				0.026	0.039	17.9	0.8974	0.015		0.005		32.4
2022 (101)	Rosana				0.044	0.040	17.2	0.9771	0.028		0.003		52.5
	Selena				0.037	0.039	17.8	0.9466	0.027		0.002		52.5
2022 (84)	Rosana	Tebuconazole	14	0.3	0.375	0.073	9.5	0.9811	0.135	0.090	0.037	26.0	66.0
	Selena				0.386	0.065	10.7	0.9694	0.155		0.021	29.9	75.0
2023 (100)	Rosana				0.370	0.080	8.7	0.9877	0.121		0.006	23.6	60.4
	Selena				0.349	0.067	10.4	0.9953	0.137		0.003	27.0	70.8

^a^ Maximum residue levels applied to apples (product code 0130010) [34]; ^b^ last applicable PHI.

**Table 6:** Dietary risk assessment for triazole residues detected in harvested apples (ranges from all trials). (A) Acute exposure (ARfD) and (B) chronic exposure (ADI) for three Czech population classes.

**(A)**	**ARfD**	**Residue at Harvest (Range)**	**Other Children**	**Adolescents**	**Adults**
	**mg/kg** **bw**	**mg/kg**	**%ARfD**	**%ARfD**	**%ARfD**
difenoconazole	0.16	0.003–0.038	0.11–1.37	0.07–0.92	0.04–0.49
mefentrifluconazole	0.15	0.006–0.027	0.21–1.04	0.14–0.69	0.08–0.37
myclobutanil	0.31	0.023–0.048	0.42–0.89	0.29–0.60	0.15–0.32
penconazole	0.5	0.004–0.010	0.05–0.12	0.03–0.08	0.02–0.04
tebuconazole *	0.03	0.003–0.162	0.48–31.09	0.32–20.84	0.17–11.12
tetraconazole	0.05	0.002–0.008	0.23–0.92	0.16–0.62	0.08–0.33
**(B)**	**ADI**	**Residue at Harvest (Range)**	**Other Children**	**Adolescents**	**Adults**
	**mg/kg** **bw/day**	**mg/kg**	**%ADI**	**%ADI**	**%ADI**
difenoconazole	0.01	0.003–0.038	0.72–9.10	0.32–4.11	0.17–2.11
mefentrifluconazole	0.035	0.006–0.027	0.38–1.85	0.17–0.83	0.09–0.43
myclobutanil	0.025	0.023–0.048	2.21–4.61	0.99–2.07	0.51–1.07
penconazole	0.03	0.004–0.010	0.32–0.80	0.14–0.36	0.08–0.19
tebuconazole *	0.03	0.003–0.162	0.20–12.92	0.09–5.82	0.05–3.02
tetraconazole	0.004	0.002–0.008	0.81–3.25	0.39–1.54	0.22–0.86

Czech population classes—other children: 3–10 years old, adolescents: 10–18 years old, adults: 18–65 years old; consumption data of apples in Czechia seen in Table 4, * residues of tebuconazole in 2020 were not included in the risk assessment as they were harvested 4 days before the end of the PHI.

## Data Availability

The original contributions presented in this study are included in the article/Supplementary Materials. Further inquiries can be directed to the corresponding author.

## Supplementary Materials

The following supporting information can be downloaded at: https://www.mdpi.com/article/10.3390/foods14183210/s1, Figure S1: Weather conditions during the field trials in 2020; Figure S2: Weather conditions during the field trials in 2021; Figure S3: Weather conditions during the field trials in 2022; Figure S4: Weather conditions during the field trials in 2023; Table S1: Fungicide products, manufacturers, pre-harvest intervals and registration in in Czechia against apple tree diseases; Table S2: The list of reagents used for laboratory analyses; Table S3: Triazole residue levels (mg/kg) and the %MRL values from the first sampling date to the harvest in 2020; Table S4: Triazole residue levels (mg/kg) and the %MRL values from the first sampling date to the harvest in 2021; Table S5: Triazole residue levels (mg/kg) and the %MRL values from the first sampling date to the harvest in 2022; Table S6: Triazole residue levels (mg/kg) and the %MRL values from the first sampling date to the harvest in 2023; Table S7: Dietary risk assessment of tested triazoles in harvested apples. (A) Acute exposure (ARfD) and (B) chronic exposure (ADI) for Czech population classes: other children, adolescents and adults.
