# Supplementary material for: Dissipation of Triazole Fungicides in Apples
Source: Foods. 2025 Sep 15;14(18):3210. doi: 10.3390/foods14183210 (PMC12469479; doi:10.3390/foods14183210)
Supplement: Supplementary file 1 [file foods-14-03210-s001.zip › foods-3848984-supplementary.pdf]

Foods

# Dissipation of Triazole Fungicides in Apples

**Tereza Horska [1], Jitka Stara [1], Frantisek Kocourek [1], Leos, Uttl [2], Jingwen Han [2], Vladimir Kocourek [2], Jana Hajslova [2], Zuzana Hanackova [3] and Dana Schusterova [2,*]**

[1] Czech Agrifood Research Center, Drnovska 507/73, 161 06 Prague, Czech Republic; tereza.horska@carc.cz (T.H.)
[2] Department of Food Analysis and Nutrition, Faculty of Food and Biochemical Technology, University of Chemistry and Technology, Prague, Technicka 1903/3, 166 28 Prague, Czech Republic
[3] Research and Breeding Institute of Pomology Holovousy Ltd., Holovousy 129, 508 01 Holovousy, Czech Republic
* Correspondence: dana.schusterova@vscht.cz

# Supplementary Material

## Figures S1–S4

## Tables S1–S7

**FIGURE CAPTIONS:**

**Figure S1.** Weather conditions during the field trials in 2020.

**Figure S2.** Weather conditions during the field trials in 2021.

**Figure S3.** Weather conditions during the field trials in 2022.

**Figure S4.** Weather conditions during the field trials in 2023.

**TABLE CAPTIONS:**

**Table S1.** Fungicide products, manufacturers, pre-harvest intervals and registration in in Czechia against apple tree diseases.

**Table S2.** The list of reagents used for laboratory analyses

**Table S3.** Triazoles residue levels (mg/kg) and the %MRL values from the first sampling date to the harvest in 2020

**Table S4.** Triazoles residue levels (mg/kg) and the %MRL values from the first sampling date to the harvest in 2021

**Table S5.** Triazoles residue levels (mg/kg) and the %MRL values from the first sampling date to the harvest in 2022

**Table S6.** Triazoles residue levels (mg/kg) and the %MRL values from the first sampling date to the harvest in 2023

**Table S7.** Dietary risk assessment of tested triazoles in harvested apples. A) Acute exposure (ARfD) and B) chronic exposure (ADI) for Czech population classes: other children, adolescents, and adults.

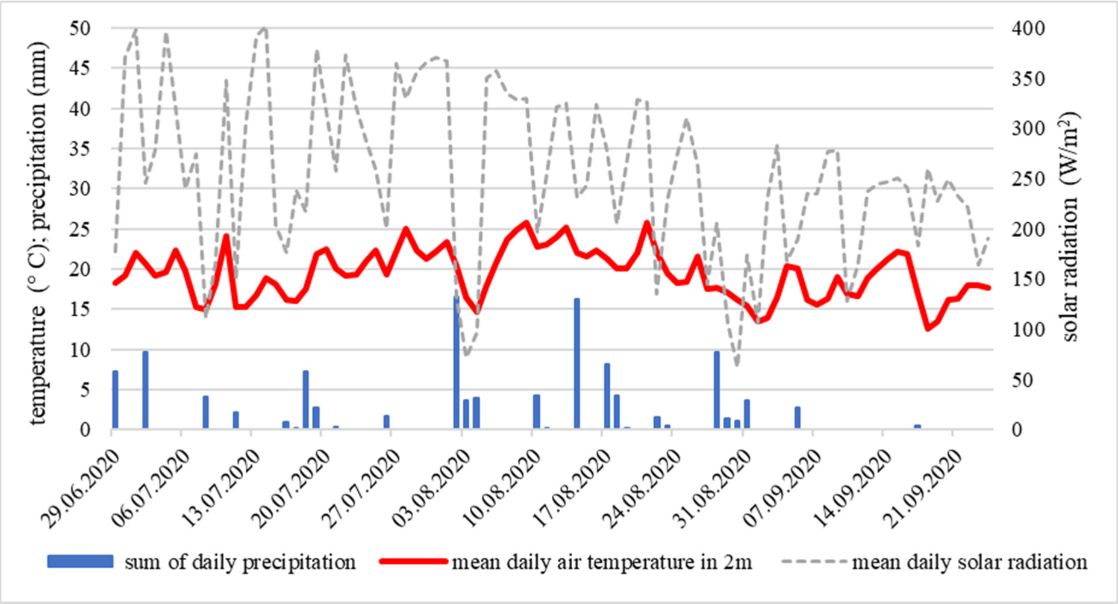


**Figure S1.** Weather conditions during the field trials in 2020.

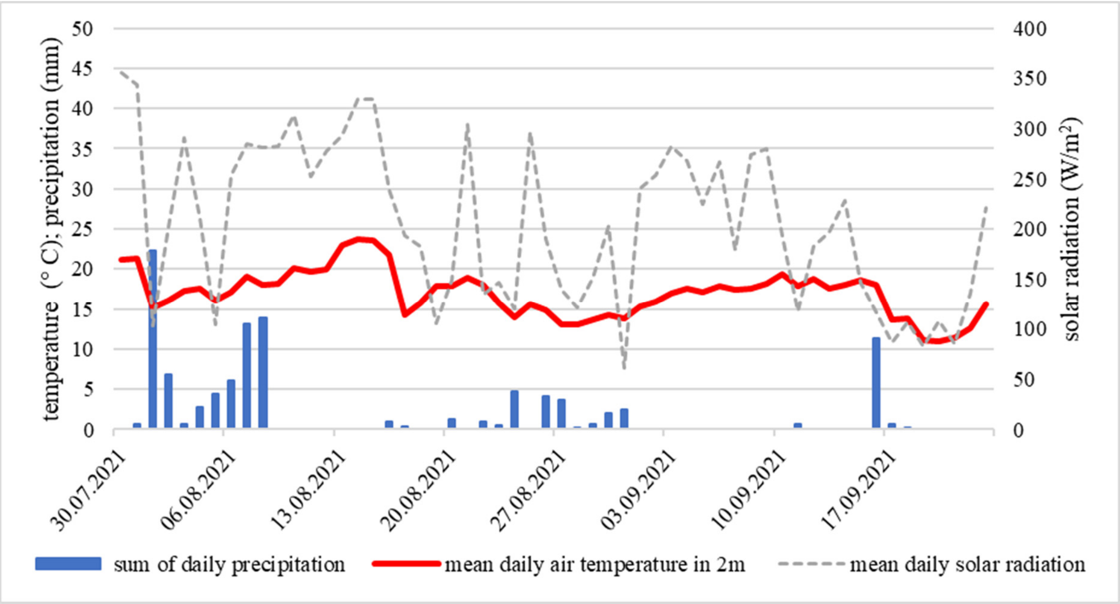


**Figure S2.** Weather conditions during the field trials in 2021.

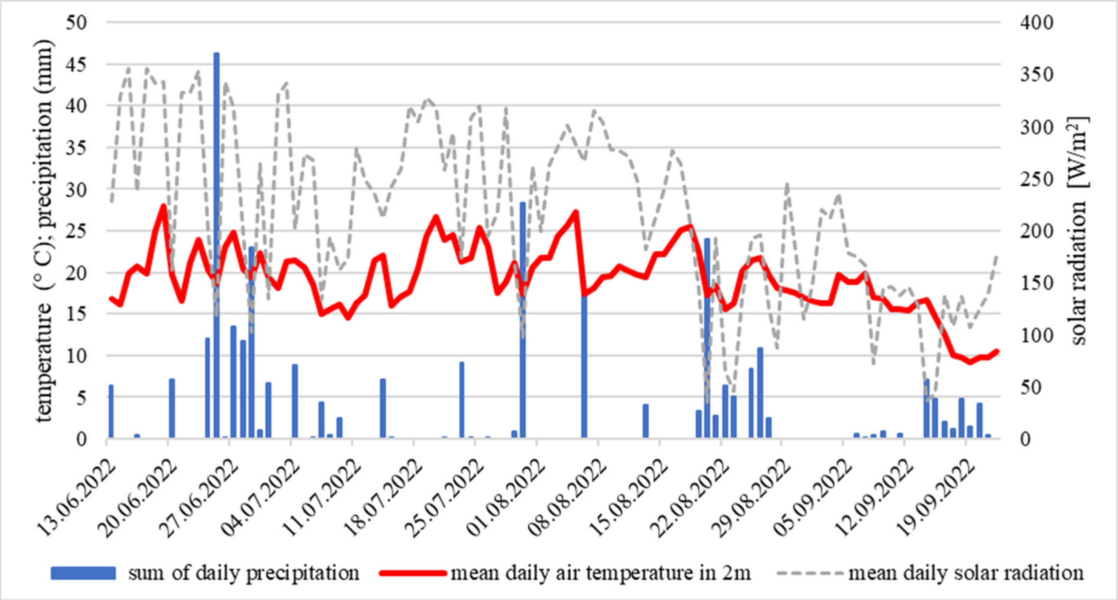


**Figure S3.** Weather conditions during the field trials in 2022.

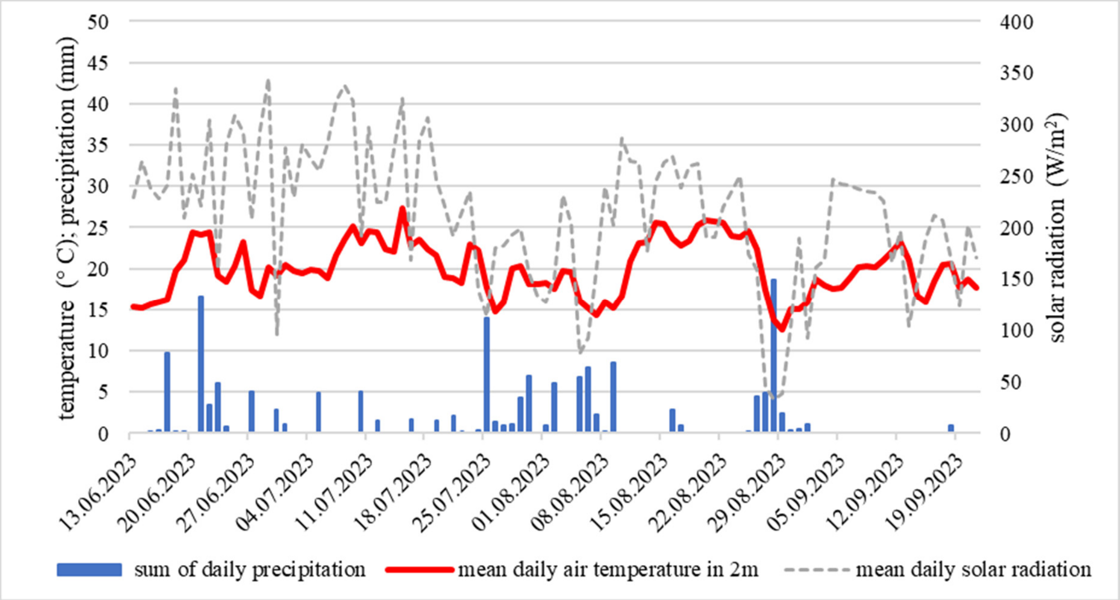


**Figure S4.** Weather conditions during the field trials in 2023

**Table S1.** Fungicide products, manufacturers, pre-harvest intervals and registration in in Czechia against apple tree diseases.

| trade name of PPP | manufacturer of PPP | PHI (days) | fungal diseases |
|---|---|---|---|
| Embrelia | Adama CZ Ltd. | 21 [a] | powdery mildew; apple scab |
| Score 250 EC | Syngenta Crop Protection AG | 49 | apple scab |
| Difol | GLOBACHEM N.V. | 110 | apple scab |
| Belanty | BASF SE | 28 | powdery mildew; apple scab |
| Topas 100 EC | Syngenta Crop Protection AG | 14 | powdery mildew |
| | | 35 | apple scab |
| Domark 10 EC | Gowan Crop Protection Limited | 14 | powdery mildew; apple scab |
| Luna Experience | Bayer S.A.S. | 14 | powdery mildew; apple scab; storage diseases |
| Talent | Corteva Agriscience Czech Ltd. | 14 [a] | powdery mildew; apple scab |

[a] previously valid pre-harvest interval

**Reference:**

Central Institute for Supervising and Testing in Agriculture. 2025. Authorised plant protection products. https://mze.gov.cz/public/app/eagriapp/por/Vyhledavani.aspx. (Accessed 03/04/2025)

**Table S2**. The list of reagents used for laboratory analyses**.**

| **reagents** | **supplier / manufacturer** |
|---|---|
| methanol (LC–MS grade)<br>ammonium formate (LC–MS grade)<br>acetonitrile (HPLC grade)<br>formic acid (99 %) | Merck KGaA (Darmstadt, Germany) |
| sodium chloride (p.a.) | Lach-ner Ltd. (Neratovice, Czechia) |
| acetone (p.a.) | Penta (Chrudim, Czechia) |
| anhydrous magnesium sulphate (p.a.) | Honeywell Fluka™ (Charlotte, NC, USA) |
| **certified analytical and internal standards** | **supplier / manufacturer** |
| difenoconazole<br>myclobutanil<br>tebuconazole | Sigma-Aldrich (Darmstadt, Germany) |
| mefentrifluconazole | HPC standards GmbH (Cunnersdorf, Germany) |
| penconazole<br>tetraconazole | Dr. Ehrenstorfer GmbH (Augsburg, Germany) |
| triphenyl phosphate (internal standard) | Sigma-Aldrich (Darmstadt, Germany) |

**Table S3.** Triazoles residue levels (mg/kg) and the %MRL values from the first sampling date to the harvest in 2020.

| sampling date | days after treatment | cultivar | residues (mg/kg) | %MRL | residues (mg/kg) | %MRL | residues (mg/kg) | %MRL |
|---|---|---|---|---|---|---|---|---|
| | | | tetraconazole | | | | | |
| 02.07.2020 | 3 | R | 0.040 | 13.3 | | | | |
| 13.07.2020 | 14 | R | 0.022 | 7.3 | | | | |
| 03.08.2020 | 35 | R | 0.016 | 5.3 | | | | |
| 14.08.2020 | 46 | R | 0.008 | 2.7 | | | | |
| 25.08.2020 | 57 | R | 0.002 | 0.7 | | | | |
| 07.09.2020 | 70 | R | 0.001 | 0.3 | | | | |
| 17.09.2020 | 80 | R | 0.002 | 0.7 | | | | |
| 24.09.2020 | 87 | R | 0.003 | 1.0 | | | | |
| 02.07.2020 | 3 | S | 0.033 | 11.0 | | | | |
| 13.07.2020 | 14 | S | 0.026 | 8.7 | | | | |
| 03.08.2020 | 35 | S | 0.012 | 4.0 | | | | |
| 14.08.2020 | 46 | S | 0.004 | 1.3 | | | | |
| 25.08.2020 | 57 | S | 0.007 | 2.3 | | | | |
| 07.09.2020 | 70 | S | 0.006 | 2.0 | | | | |
| 17.09.2020 | 80 | S | 0.004 | 1.3 | | | | |
| 24.09.2020 | 87 | S | 0.005 | 1.7 | | | | |
| | | | difenoconazole | | penconazole | | myclobutanil | |
| 03.08.2020 | 3 | R | 0.067 | 8.4 | 0.054 | 36.0 | 0.130 | 21.7 |
| 14.08.2020 | 14 | R | 0.027 | 3.4 | 0.014 | 9.3 | 0.044 | 7.3 |
| 25.08.2020 | 25 | R | 0.025 | 3.1 | 0.009 | 6.0 | 0.043 | 7.2 |
| 07.09.2020 | 38 | R | 0.013 | 1.6 | 0.006 | 4.0 | 0.032 | 5.3 |
| 17.09.2020 | 48 | R | 0.015 | 1.9 | 0.005 | 3.3 | 0.032 | 5.3 |
| 24.09.2020 | 55 | R | 0.015 | 1.9 | 0.004 | 2.7 | 0.032 | 5.3 |
| 03.08.2020 | 3 | S | 0.050 | 6.3 | 0.041 | 27.3 | 0.127 | 21.2 |
| 14.08.2020 | 14 | S | 0.026 | 3.3 | 0.015 | 10.0 | 0.047 | 7.8 |
| 25.08.2020 | 25 | S | 0.027 | 3.4 | 0.009 | 6.0 | 0.043 | 7.2 |
| 07.09.2020 | 38 | S | 0.007 | 0.9 | 0.002 | 1.3 | 0.012 | 2.0 |
| 17.09.2020 | 48 | S | 0.012 | 1.5 | 0.004 | 2.7 | 0.012 | 2.0 |
| 24.09.2020 | 55 | S | 0.016 | 2.0 | 0.004 | 2.7 | 0.023 | 3.8 |
| | | | tebuconazole | | | | | |
| 17.09.2020 | 3 | R | 0.244 | 81.3 | | | | |
| 24.09.2020 | 10 | R | 0.227 | 75.7 | | | | |
| 17.09.2020 | 3 | S | 0.169 | 56.3 | | | | |
| 24.09.2020 | 10 | S | 0.217 | 72.3 | | | | |

**Table S4.** Triazoles residue levels (mg/kg) and the %MRL values from the first sampling date to the harvest in 2021.

| sampling date | days after treatment | cultivar | residues (mg/kg) | %MRL | residues (mg/kg) | %MRL | residues (mg/kg) | %MRL |
|---|---|---|---|---|---|---|---|---|
| | | | tetraconazole | | | | | |
| 02.08.2021 | 3 | R | 0.023 | 7.7 | | | | |
| 13.08.2021 | 14 | R | 0.013 | 4.3 | | | | |
| 25.08.2021 | 25 | R | 0.009 | 3.0 | | | | |
| 06.09.2021 | 38 | R | 0.013 | 4.3 | | | | |
| 16.09.2021 | 48 | R | 0.003 | 1.0 | | | | |
| 23.09.2021 | 55 | R | 0.008 | 2.7 | | | | |
| 02.08.2021 | 3 | S | 0.024 | 8.0 | | | | |
| 13.08.2021 | 14 | S | 0.014 | 4.7 | | | | |
| 25.08.2021 | 25 | S | 0.006 | 2.0 | | | | |
| 06.09.2021 | 38 | S | 0.009 | 3.0 | | | | |
| 16.09.2021 | 48 | S | 0.004 | 1.3 | | | | |
| 23.09.2021 | 55 | S | 0.005 | 1.7 | | | | |
| | | | difenoconazole | | penconazole | | myclobutanil | |
| 13.08.2021 | 3 | R | 0.071 | 8.9 | 0.057 | 38.0 | 0.147 | 24.5 |
| 25.08.2021 | 15 | R | 0.050 | 6.3 | 0.018 | 12.0 | 0.041 | 6.8 |
| 06.09.2021 | 27 | R | 0.040 | 5.0 | 0.012 | 8.0 | 0.069 | 11.5 |
| 16.09.2021 | 37 | R | 0.043 | 5.4 | 0.012 | 8.0 | 0.065 | 10.8 |
| 23.09.2021 | 44 | R | 0.038 | 4.8 | 0.010 | 6.7 | 0.048 | 8.0 |
| 13.08.2021 | 3 | S | 0.110 | 13.8 | 0.055 | 36.7 | 0.132 | 22.0 |
| 25.08.2021 | 15 | S | 0.060 | 7.5 | 0.020 | 13.3 | 0.044 | 7.3 |
| 06.09.2021 | 27 | S | 0.036 | 4.5 | 0.012 | 8.0 | 0.062 | 10.3 |
| 16.09.2021 | 37 | S | 0.033 | 4.1 | 0.010 | 6.7 | 0.045 | 7.5 |
| 23.09.2021 | 44 | S | 0.027 | 3.4 | 0.007 | 4.7 | 0.039 | 6.5 |
| | | | tebuconazole | | | | | |
| 06.09.2021 | 3 | R | 0.210 | 70.0 | | | | |
| 16.09.2021 | 13 | R | 0.230 | 76.7 | | | | |
| 23.09.2021 | 20 | R | 0.162 | 54.0 | | | | |
| 06.09.2021 | 3 | S | 0.290 | 96.7 | | | | |
| 16.09.2021 | 13 | S | 0.200 | 66.7 | | | | |
| 23.09.2021 | 20 | S | 0.148 | 49.3 | | | | |

**Table S5.** Triazoles residue levels (mg/kg) and the %MRL values from the first sampling date to the harvest in 2022.

| sampling date | days after treatment | cultivar | residues (mg/kg) | %MRL | residues (mg/kg) | %MRL | residues (mg/kg) | %MRL |
|---|---|---|---|---|---|---|---|---|
| | | | tetraconazole | | | | | |
| 16.06.2022 | 3 | R | 0.045 | 15.0 | | | | |
| 01.07.2022 | 18 | R | 0.020 | 6.7 | | | | |
| 14.07.2022 | 31 | R | 0.014 | 4.7 | | | | |
| 28.07.2022 | 45 | R | 0.009 | 3.0 | | | | |
| 12.08.2022 | 60 | R | 0.007 | 2.3 | | | | |
| 01.09.2022 | 80 | R | 0.003 | 1.0 | | | | |
| 22.09.2022 | 101 | R | 0.003 | 1.0 | | | | |
| 16.06.2022 | 3 | S | 0.042 | 14.0 | | | | |
| 01.07.2022 | 18 | S | 0.023 | 7.7 | | | | |
| 14.07.2022 | 31 | S | 0.008 | 2.7 | | | | |
| 28.07.2022 | 45 | S | 0.011 | 3.7 | | | | |
| 12.08.2022 | 60 | S | 0.009 | 3.0 | | | | |
| 01.09.2022 | 80 | S | 0.002 | 0.7 | | | | |
| 22.09.2022 | 101 | S | 0.002 | 0.7 | | | | |
| | | | difenoconazole | | mefentrifluconazole | | tebuconazole | |
| 01.07.2022 | 1 | R | 0.100 | 12.5 | 0.370 | 92.5 | 0.350 | 116.7 |
| 14.07.2022 | 14 | R | 0.050 | 6.3 | 0.110 | 27.5 | 0.130 | 43.3 |
| 28.07.2022 | 28 | R | 0.009 | 1.1 | 0.035 | 8.8 | 0.045 | 15.0 |
| 12.08.2022 | 43 | R | 0.004 | 0.5 | 0.020 | 5.0 | 0.027 | 9.0 |
| 01.09.2022 | 63 | R | 0.002 | 0.3 | 0.008 | 2.0 | 0.014 | 4.7 |
| 22.09.2022 | 84 | R | 0.006 | 0.8 | 0.027 | 6.8 | 0.037 | 12.3 |
| 01.07.2022 | 1 | S | 0.100 | 12.5 | 0.330 | 82.5 | 0.370 | 123.3 |
| 14.07.2022 | 14 | S | 0.031 | 3.9 | 0.133 | 33.3 | 0.127 | 42.3 |
| 28.07.2022 | 28 | S | 0.013 | 1.6 | 0.056 | 14.0 | 0.072 | 24.0 |
| 12.08.2022 | 43 | S | 0.012 | 1.5 | 0.038 | 9.5 | 0.060 | 20.0 |
| 01.09.2022 | 63 | S | 0.002 | 0.3 | 0.011 | 2.8 | 0.014 | 4.7 |
| 22.09.2022 | 84 | S | 0.003 | 0.4 | 0.015 | 3.8 | 0.021 | 7.0 |

**Table S6.** Triazoles residue levels (mg/kg) and the %MRL values from the first sampling date to the harvest in 2023**.**

| sampling date | days after treatment | cultivar | residues (mg/kg) | %MRL | residues (mg/kg) | %MRL | residues (mg/kg) | %MRL |
|---|---|---|---|---|---|---|---|---|
| | | | difenoconazole | | mefentrifluconazole | | tebuconazole | |
| 14.06.2023 | 1 | R | 0.063 | 7.9 | 0.318 | 79.4 | 0.345 | 115.0 |
| 27.06.2023 | 14 | R | 0.011 | 1.4 | 0.081 | 20.3 | 0.102 | 33.8 |
| 10.07.2023 | 27 | R | 0.007 | 0.9 | 0.049 | 12.1 | 0.069 | 22.8 |
| 24.07.2023 | 41 | R | 0.001 | 0.1 | 0.013 | 3.3 | 0.016 | 5.3 |
| 10.08.2023 | 58 | R | 0.001 | 0.1 | 0.006 | 1.5 | 0.008 | 2.5 |
| 31.08.2023 | 79 | R | <0.001 | <0.1 | 0.005 | 1.1 | 0.007 | 2.3 |
| 15.09.2023 | 94 | R | <0.001 | <0.1 | 0.005 | 1.1 | 0.006 | 2.0 |
| 21.09.2023 | 100 | R | <0.001 | <0.1 | 0.006 | 1.4 | 0.006 | 2.0 |
| 14.06.2023 | 1 | S | 0.056 | 7.0 | 0.280 | 70.0 | 0.330 | 110.0 |
| 27.06.2023 | 14 | S | 0.011 | 1.4 | 0.094 | 23.4 | 0.123 | 41.0 |
| 10.07.2023 | 27 | S | 0.006 | 0.7 | 0.053 | 13.3 | 0.070 | 23.2 |
| 24.07.2023 | 41 | S | 0.002 | 0.3 | 0.027 | 6.8 | 0.027 | 8.8 |
| 10.08.2023 | 58 | S | 0.001 | 0.1 | 0.011 | 2.6 | 0.012 | 4.0 |
| 31.08.2023 | 79 | S | <0.001 | <0.1 | 0.007 | 1.8 | 0.006 | 2.0 |
| 15.09.2023 | 94 | S | <0.001 | <0.1 | 0.005 | 1.3 | 0.004 | 1.2 |
| 21.09.2023 | 100 | S | <0.001 | <0.1 | 0.006 | 1.5 | 0.003 | 0.8 |
| | | | penconazole | | | | | |
| 27.06.2023 | 1 | R | 0.045 | 30.0 | | | | |
| 10.07.2023 | 14 | R | 0.005 | 3.0 | | | | |
| 24.07.2023 | 28 | R | 0.001 | 0.7 | | | | |
| 10.08.2023 | 45 | R | 0.001 | 0.7 | | | | |
| 31.08.2023 | 66 | R | <0.001 | <0.7 | | | | |
| 15.09.2023 | 81 | R | <0.001 | <0.7 | | | | |
| 21.09.2023 | 87 | R | <0.001 | <0.7 | | | | |
| 27.06.2023 | 1 | S | 0.050 | 33.0 | | | | |
| 10.07.2023 | 14 | S | 0.008 | 5.0 | | | | |
| 24.07.2023 | 28 | S | 0.003 | 1.7 | | | | |
| 10.08.2023 | 45 | S | 0.001 | 0.7 | | | | |
| 31.08.2023 | 66 | S | <0.001 | <0.7 | | | | |
| 15.09.2023 | 81 | S | <0.001 | <0.7 | | | | |
| 21.09.2023 | 87 | S | <0.001 | <0.7 | | | | |

**Table S7.** Dietary risk assessment of tested triazoles in harvested apples. **A**) Acute exposure (ARfD) and **B**) chronic exposure (ADI) for Czech population classes: other children, adolescents, and adults.

**A)**

| year (trial PHI) | cultivar | active ingredient | residue at harvest | ARfD | other children exposure | | adolescent exposure | | adult exposure | |
|---|---|---|---|---|---|---|---|---|---|---|
| | | | mg/kg | mg/kg bw | µg/kg bw | %ARfD | µg/kg bw | %ARfD | µg/kg bw | %ARfD |
| 2020 | Rosana | | 0.015 | 0.16 | 0.864 | 0.54 | 0.579 | 0.36 | 0.308 | 0.19 |
| (55) | Selena | difenoconazole | 0.016 | 0.16 | 0.922 | 0.58 | 0.618 | 0.39 | 0.329 | 0.21 |
| 2021 | Rosana | (Embrelia) | 0.038 | 0.16 | 2.189 | 1.37 | 1.467 | 0.92 | 0.781 | 0.49 |
| (44) | Selena | | 0.027 | 0.16 | 1.555 | 0.97 | 1.042 | 0.65 | 0.555 | 0.35 |
| 2022 | Rosana | difenoconazole | 0.006 | 0.16 | 0.346 | 0.22 | 0.232 | 0.15 | 0.123 | 0.08 |
| (84) | Selena | (Score 250 EC) | 0.003 | 0.16 | 0.173 | 0.11 | 0.116 | 0.07 | 0.062 | 0.04 |
| 2023 | Rosana | difenoconazole | <0.001 | 0.16 | - | - | - | - | - | - |
| (100) | Selena | (Difol) | <0.001 | 0.16 | - | - | - | - | - | - |
| 2022 | Rosana | | 0.027 | 0.15 | 1.555 | 1.04 | 1.042 | 0.69 | 0.555 | 0.37 |
| (84) | Selena | | 0.015 | 0.15 | 0.864 | 0.58 | 0.579 | 0.38 | 0.308 | 0.21 |
| 2023 | Rosana | mefentrifluconazole | 0.006 | 0.15 | 0.317 | 0.21 | 0.212 | 0.14 | 0.113 | 0.08 |
| (100) | Selena | | 0.006 | 0.15 | 0.346 | 0.23 | 0.232 | 0.15 | 0.123 | 0.08 |
| 2020 | Rosana | | 0.032 | 0.31 | 1.843 | 0.59 | 1.235 | 0.40 | 0.658 | 0.21 |
| (55) | Selena | | 0.023 | 0.31 | 1.325 | 0.42 | 0.888 | 0.29 | 0.473 | 0.15 |
| 2021 | Rosana | myclobutanil | 0.048 | 0.31 | 2.765 | 0.89 | 1.853 | 0.60 | 0.987 | 0.32 |
| (44) | Selena | | 0.039 | 0.31 | 2.246 | 0.72 | 1.505 | 0.49 | 0.802 | 0.26 |
| 2020 | Rosana | | 0.004 | 0.5 | 0.230 | 0.05 | 0.154 | 0.03 | 0.082 | 0.02 |
| (55) | Selena | | 0.004 | 0.5 | 0.230 | 0.05 | 0.154 | 0.03 | 0.082 | 0.02 |
| 2021 | Rosana | penconazole | 0.010 | 0.5 | 0.576 | 0.12 | 0.386 | 0.08 | 0.206 | 0.04 |
| (44) | Selena | | 0.007 | 0.5 | 0.403 | 0.08 | 0.270 | 0.06 | 0.144 | 0.03 |
| | Rosana | | <0.001 | 0.5 | - | - | - | - | - | - |

| | | | | | | | | | | |
|---|---|---|---|---|---|---|---|---|---|---|
| 2023 (87) | Selena | | <0.001 | 0.5 | - | - | - | - | - | - |
| 2020 | Rosana | tebuconazole | 0.227 | 0.03 | 13.074 | 43.56 | 8.761 | 29.20 | 4.669 | 15.58 |
| (10) | Selena | | 0.217 | 0.03 | 12.498 | 41.64 | 8.375 | 27.92 | 4.463 | 14.90 |
| 2021 | Rosana | | 0.162 | 0.03 | 9.330 | 31.09 | 6.252 | 20.84 | 3.332 | 11.12 |
| (20) | Selena | | 0.148 | 0.03 | 8.524 | 28.40 | 5.712 | 19.04 | 3.044 | 10.16 |
| 2022 | Rosana | | 0.037 | 0.03 | 2.131 | 7.10 | 1.428 | 4.76 | 0.761 | 2.54 |
| (84) | Selena | | 0.021 | 0.03 | 1.209 | 4.03 | 0.810 | 2.70 | 0.432 | 1.44 |
| 2023 | Rosana | | 0.006 | 0.03 | 0.346 | 1.15 | 0.232 | 0.77 | 0.123 | 0.41 |
| (100) | Selena | | 0.003 | 0.03 | 0.144 | 0.48 | 0.096 | 0.32 | 0.051 | 0.17 |
| 2020 | Rosana | tetraconazole | 0.003 | 0.05 | 0.173 | 0.35 | 0.116 | 0.23 | 0.062 | 0.12 |
| (87) | Selena | | 0.005 | 0.05 | 0.288 | 0.58 | 0.193 | 0.39 | 0.103 | 0.21 |
| 2021 | Rosana | | 0.008 | 0.05 | 0.461 | 0.92 | 0.309 | 0.62 | 0.165 | 0.33 |
| (55) | Selena | | 0.005 | 0.05 | 0.288 | 0.58 | 0.193 | 0.39 | 0.103 | 0.21 |
| 2022 | Rosana | | 0.003 | 0.05 | 0.173 | 0.35 | 0.116 | 0.23 | 0.062 | 0.12 |
| (101) | Selena | | 0.002 | 0.05 | 0.115 | 0.23 | 0.077 | 0.16 | 0.041 | 0.08 |

**B)**

| year (trial PHI) | cultivar | active ingredient | residue at harvest | ADI | other children exposure | | adolescent exposure | | adult exposure | |
|---|---|---|---|---|---|---|---|---|---|---|
| | | | mg/kg | μg/kg bw/day | μg/kg bw/day | %ADI | μg/kg bw/day | %ADI | μg/kg bw/day | %ADI |
| 2020 | Rosana | difenoconazole (Embrelia) | 0.015 | 0.01 | 0.359 | 3.59 | 0.162 | 1.62 | 0.083 | 0.83 |
| (55) | Selena | | 0.016 | 0.01 | 0.383 | 3.83 | 0.173 | 1.73 | 0.089 | 0.89 |
| 2021 | Rosana | | 0.038 | 0.01 | 0.910 | 9.10 | 0.411 | 4.11 | 0.211 | 2.11 |
| (44) | Selena | | 0.027 | 0.01 | 0.647 | 6.47 | 0.292 | 2.92 | 0.150 | 1.50 |
| 2022 | Rosana | difenoconazole (Score 250 EC) | 0.006 | 0.01 | 0.144 | 1.44 | 0.065 | 0.65 | 0.033 | 0.33 |
| (84) | Selena | | 0.003 | 0.01 | 0.072 | 0.72 | 0.032 | 0.32 | 0.017 | 0.17 |
| 2023 | Rosana | difenoconazole (Difol) | <0.001 | 0.01 | - | - | - | - | - | - |
| (100) | Selena | | <0.001 | 0.01 | - | - | - | - | - | - |
| 2022 | Rosana | mefentrifluconazole | 0.027 | 0.035 | 0.647 | 1.85 | 0.292 | 0.83 | 0.150 | 0.43 |
| (84) | Selena | | 0.015 | 0.035 | 0.359 | 1.03 | 0.162 | 0.46 | 0.083 | 0.24 |
| 2023 | Rosana | | 0.006 | 0.035 | 0.132 | 0.38 | 0.059 | 0.17 | 0.031 | 0.09 |
| (100) | Selena | | 0.006 | 0.035 | 0.144 | 0.41 | 0.065 | 0.18 | 0.033 | 0.10 |
| 2020 | Rosana | myclobutanil | 0.032 | 0.025 | 0.766 | 3.07 | 0.346 | 1.38 | 0.178 | 0.71 |
| (55) | Selena | | 0.023 | 0.025 | 0.551 | 2.21 | 0.249 | 0.99 | 0.128 | 0.51 |
| 2021 | Rosana | | 0.048 | 0.025 | 1.149 | 4.61 | 0.519 | 2.07 | 0.267 | 1.07 |
| (44) | Selena | | 0.039 | 0.025 | 0.934 | 3.74 | 0.422 | 1.68 | 0.217 | 0.87 |
| 2020 | Rosana | penconazole | 0.004 | 0.03 | 0.096 | 0.32 | 0.043 | 0.14 | 0.022 | 0.08 |
| (55) | Selena | | 0.004 | 0.03 | 0.096 | 0.32 | 0.043 | 0.14 | 0.022 | 0.08 |
| 2021 | Rosana | | 0.010 | 0.03 | 0.239 | 0.80 | 0.108 | 0.36 | 0.056 | 0.19 |
| (44) | Selena | | 0.007 | 0.03 | 0.167 | 0.56 | 0.076 | 0.25 | 0.039 | 0.13 |
| 2023 | Rosana | | <0.001 | 0.03 | - | - | - | - | - | - |
| (87) | Selena | | <0.001 | 0.03 | - | - | - | - | - | - |
| | Rosana | tebuconazole | 0.227 | 0.03 | 5.436 | 18.1 | 2.454 | 8.2 | 1.264 | 4.2 |

| | | | | | | | | | | |
|---|---|---|---|---|---|---|---|---|---|---|
| 2020 (10) | Selena | | 0.217 | 0.03 | 5.196 | 17.3 | 2.346 | 7.8 | 1.208 | 4.0 |
| 2021 | Rosana | | 0.162 | 0.03 | 3.879 | 12.9 | 1.751 | 5.8 | 0.902 | 3.0 |
| (20) | Selena | | 0.148 | 0.03 | 3.544 | 11.8 | 1.600 | 5.3 | 0.824 | 2.8 |
| 2022 | Rosana | | 0.037 | 0.03 | 0.886 | 2.95 | 0.400 | 1.33 | 0.206 | 0.69 |
| (84) | Selena | | 0.021 | 0.03 | 0.503 | 1.67 | 0.227 | 0.75 | 0.117 | 0.39 |
| 2023 | Rosana | | 0.006 | 0.03 | 0.144 | 0.48 | 0.065 | 0.22 | 0.033 | 0.11 |
| (100) | Selena | | 0.003 | 0.03 | 0.060 | 0.20 | 0.027 | 0.09 | 0.014 | 0.05 |
| 2020 | Rosana | tetraconazole | 0.003 | 0.004 | 0.049 | 1.22 | 0.023 | 0.58 | 0.013 | 0.32 |
| (87) | Selena | | 0.005 | 0.004 | 0.081 | 2.03 | 0.039 | 0.96 | 0.021 | 0.54 |
| 2021 | Rosana | | 0.008 | 0.004 | 0.130 | 3.25 | 0.062 | 1.54 | 0.034 | 0.86 |
| (55) | Selena | | 0.005 | 0.004 | 0.081 | 2.03 | 0.039 | 0.96 | 0.021 | 0.54 |
| 2022 | Rosana | | 0.003 | 0.004 | 0.049 | 1.22 | 0.023 | 0.58 | 0.013 | 0.32 |
| (101) | Selena | | 0.002 | 0.004 | 0.033 | 0.81 | 0.016 | 0.39 | 0.009 | 0.22 |
